# The search for a unifying diagnosis involving neurological, endocrine and immune dysfunction: a case report of a novel presentation of DAVID syndrome

**DOI:** 10.1186/s12887-022-03760-x

**Published:** 2022-12-09

**Authors:** M. Price, P. L. Hofman, K. Hsiao, H. F. Jones

**Affiliations:** 1 Department of Immunology, Starship Child Health, Auckland, New Zealand; 2 Department of Endocrinology, Starship Child Health, Auckland, New Zealand; 3 Liggins Institute, University of Auckland, Auckland, New Zealand; 4 Department of Paediatrics, University of Auckland, Auckland, New Zealand; 5 Department of Neuroservices, Paediatric Neuroservices, Starship Child Health, Auckland, New Zealand; 6 Centre for Brain Research, University of Auckland, Auckland, New Zealand

**Keywords:** DAVID syndrome, Addisonian crisis, Pseudotumour cerebri, Panhypogammaglobulinaemia

## Abstract

**Background:**

We report a novel presentation of deficit in anterior pituitary function with variable immune deficiency (DAVID) syndrome in a healthy young girl presenting in Addisonian crisis with raised intracranial pressure. Nearly all cases of DAVID syndrome described in the literature have presented with recurrent infections and variable immunodeficiency. Pseudotumour cerebri has not been reported in DAVID syndrome to date.

**Case presentation:**

A four-year-old girl represented to hospital with vomiting, confusion and diplopia after ten days of tiredness, neck and abdominal pain, and headache. Her cranial nerve examination demonstrated a right abducens nerve palsy and papilloedema, and she was found to have ketotic hypoglycaemia and hypocortisolaemia secondary to adrenocorticotrophic hormone (ACTH) deficiency. Her neuroimaging was consistent with pseudotumour cerebri, and her lumbar puncture opening pressure confirmed raised intracranial pressure (30–40 cmH_2_O). Cerebrospinal fluid analysis was normal. The patient’s symptoms improved with hydrocortisone replacement and acetazolamide, but the raised intracranial pressure recurred after acetazolamide was discontinued. She was subsequently found to have panhypogammaglobulinaemia, and DAVID syndrome was diagnosed. Genetic testing demonstrated a truncating mutation in the *NFKB2* gene c.2557C > T, *p*.(Arg853*).

**Conclusions:**

This case demonstrates pseudotumour cerebri as a novel neurological presentation of DAVID syndrome, highlights the rare association between adrenal insufficiency and intracranial hypertension, and shows the challenges in diagnosing isolated ACTH deficiency. We emphasise that cortisol should be checked in pre-pubertal children with pseudotumour cerebri and a diagnosis of DAVID syndrome considered in those presenting with low cortisol and neurological symptoms.

## Background

The rare association between isolated ACTH deficiency and common variable immunodeficiency (CVID) was first reported in 1991 [1], and has since been termed, ‘Deficit in anterior pituitary function with variable immune deficiency (DAVID) syndrome’ [2]. The unusual combination of CVID and ACTH deficiency is caused by mutations in the *NFKB2* gene which result in haploinsufficiency in the non-canonical NF-kB2 signaling pathway which regulates B cell maturation [3].

DAVID syndrome typically presents with recurrent infections and primary immune deficiency at two to eight years of age, followed by ACTH-deficiency, most commonly presenting with symptomatic hypoglycaemia, at five to 15 years of age [2, 3]. In a recently expanded cohort of 50 carriers of pathogenic *NFKB2* mutations, primary immune deficiency was observed in 92% and ACTH-deficiency in 44% of patients [3]. Eighty percent of patients had autoimmune manifestations with alopecia and lymphocytic infiltration of solid organs being most commonly observed [3]. Two carriers were asymptomatic. Absence of primary immune deficiency and autoimmunity in some individuals is consistent with variable clinical expressivity of loss of function mutations in the *NFKB2* gene.

Neurological manifestations described in DAVID syndrome comprise seizures secondary to hypoglycaemia [4, 5], and aseptic and infectious meningitis, usually in individuals already diagnosed with CVID [3].

We report a novel presentation of DAVID syndrome in a previously healthy young girl with rapidly evolving adrenal insufficiency and pseudotumour cerebri culminating in Addisonian crisis.

## Case presentation

### Initial presentation

A four-year-old New Zealand European/Māori girl was admitted to hospital following five days of tiredness, neck and abdominal pain, vomiting and headache. On examination, her GCS score was 15, and she had meningism but no focal neurological deficits. A lumbar puncture was performed to investigate for meningitis. Cerebrospinal fluid (CSF) parameters were within normal limits, though the opening pressure was not recorded. Electrolytes and a random glucose level were normal. Her symptoms slowly resolved and she was discharged home after five days. However she represented the following day with vomiting, confusion and diplopia.

### Examination

The patient was drowsy but able to follow commands, afebrile and persistently tachycardic. Her blood pressure was normal. Cranial nerve examination demonstrated a right abducens nerve palsy and papilloedema. Her visual fields were intact and her neurological examination was otherwise normal.

### Investigations

**Fig. 1:**
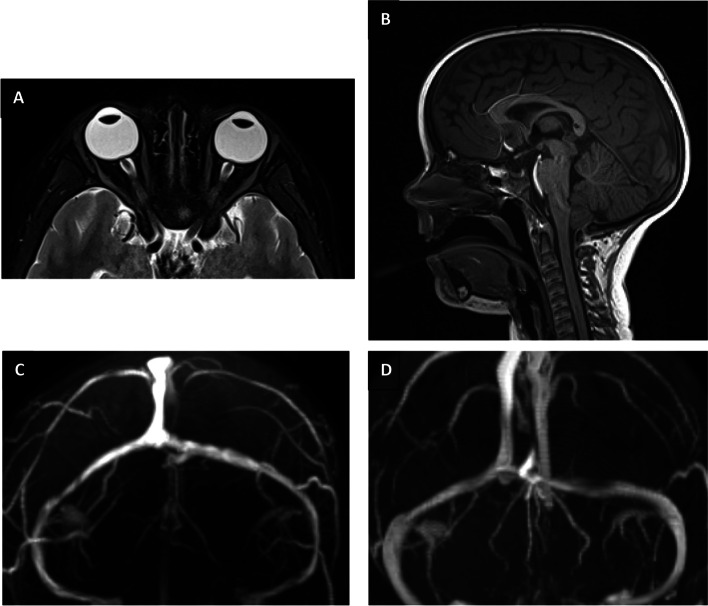
Magnetic resonance brain imaging demonstrated features consistent with intracranial hypertension. **A** T2-weighted with fat suppression axial image demonstrating flattening of the sclera of both globes posteriorly with papilloedema and distension of the optic nerve sheaths. **B** T1-weighted sagittal image demonstrating intrasellar arachnoid herniation. The pituitary gland and stalk were normal in appearance. **C** Time of flight MR venogram demonstrating bilateral stenosis of the lateral segments of the transverse sinuses, which resolves after treatment (**D**)

A glucose level of 1.9 mmol/L was noted with a beta-hydroxybutyrate level elevated at 6.1 mmol/L. A full hypoglycaemia screen was performed and hypocortisolaemia < 30 nmol/L was identified (Table 1). An ACTH level was < 1 pmol/L suggesting a central cause for the low cortisol level. Her growth hormone/IGF-1 and thyroid hormone levels were normal. Neuroimaging (CT head and MR brain) demonstrated features consistent with pseudotumour cerebri (Fig. 1). There was no evidence of dural or cavernous sinus thrombosis, Chiari malformation, or a space occupying lesion. Her CSF opening pressure was elevated at 30-40cmH_2_O, which was reduced to 20cmH_2_O during the procedure.

**Table 1:** Initial hypoglycaemia screen and CSF results

Parameter	Patient	Normal Range
**Glucose**	1.9 mmol/L	> 2.6 mmol/L
**Insulin**	< 0.4mIU/L	2.6–24.9mIU/L
**B-hydroxybutyrate**	6.37 mmol/L	0.0–0.27 mmol/L
**Free fatty acids**	2.4 mEq/L	0.0–0.6 mEq/L
**Cortisol**	< 30 nmol/L	> 400 nmol/L
**Growth hormone**	14.6ug/L	> 5ug/L
**Ammonia**	44umol/L	0.0-70umol/L
**Pyruvate**	0.06 mol/L	0.0–0.18 mol/L
**Amino acids**	Not suggestive of metabolic disease	
**CSF 1:** Initial presentation	WCC < 1, RBC < 1 0% polymorphonuclear cells, 5% lymphocytes, 1% monocytes Protein 0.23, glucose 2.6 No growth, viral PCR negative	
**CSF 2:** Eight days following presentation	WCC 2, RBC 22 8% polymorphonuclear cells, 53% lymphocytes, 39% monocytes Protein 0.23, glucose 2.8 No growth	
**CSF 3:** Three months following presentation	WCC 1, RBC < 1 Insufficient cells for differential Protein 0.28, glucose 2.9 No growth Borderline raised neopterin (31.25 nmol/L), normal amino acids, CSF IgG < 4 mg/L, Serum IgG 1.9 g/L, CSF/serum albumin index 3.3. Matching oligoclonal IgG bands present in CSF and serum	

*WCC* White cell count, *RBC* Red blood cell count

A short Synacthen test performed two days later was consistent with cortisol deficiency with a post-Synacthen cortisol of 257 nmol/L. Stimulated levels greater than 400 nmol/L are considered a normal response. A repeat ACTH level was < 1 pmol/L, indicating central adrenal insufficiency. Central ACTH suppression due to prolonged exogenous glucocorticoid administration was excluded due to lack of history and absence of urine steroid metabolites.

### Management

The patient was started on acetazolamide 12.5 mg/kg/dose BD and maintenance steroid replacement (hydrocortisone 10 mg/m^2^/day cortisol equivalence). Her symptoms and right abducens nerve palsy improved.

### Progress

A short Synacthen test was repeated one month later, after the hydrocortisone had been ceased for two weeks, and confirmed a severe central defect with a pre-Synacthen cortisol of 5 nmol/L rising to a peak of 14 nmol/L. Acetazolamide was discontinued two months later, at least two weeks after the patient’s papilloedema had resolved. However, the patient represented with diplopia, subtle recurrence of her right abducens palsy and mild papilloedema one month later. A repeat MR brain was normal and opening pressure under sedation was 26cmH_2_O. In view of her symptoms, she was recommenced on low-dose acetazolamide.

In the absence of an underlying diagnosis, further work-up included serum immunoglobulins. She was found to have panhypogammaglobinaemia (IgG 1.9 g/L, IgA 0.11 g/L, IgM 0.29 g/L). Specific questioning elicited a history of frequent mild upper respiratory tract infections (URTIs) between one and three years of age, and one episode of *Campylobacter jejuni* gastroenteritis. There was no other history or evidence of heightened infection susceptibility or autoimmunity. Further immune evaluation revealed a significant switched memory B cell lymphopenia (< 1.0%), in the setting of preserved T, B and NK cell proportions, and absent antibody responses to toxoid and conjugate vaccines, consistent with impaired B cell differentiation and function.

Given the clinical phenotype of ACTH deficiency and features of a B cell defect, deficit in anterior pituitary function with variable immune deficiency (DAVID) syndrome secondary to damaging variant(s) in candidate genes including *NFKB2, TNFRSF138, IKZF1* and *TBX19* was suspected. Next-generation-sequencing gene panel testing (Blueprint Genetics Primary Immunodeficiency Flex Panel, Version 1, 2020) confirmed the presence of a nonsense mutation in the *NFKB2* gene (NM_001077494.3 c.2557C > T, p.Arg853*). There was no family history of immune deficiency or autoimmunity, and parental gene testing is pending.

### Follow up

The patient’s blood glucose levels have remained within normal limits since starting replacement glucocorticoid therapy and her tachycardia has resolved. She remains well and infection free on immunoglobulin replacement therapy. The acetazolamide has since been stopped, without recurrence of her papilloedema.

## Discussion

We report a previously healthy child with DAVID syndrome secondary to a heterozygous nonsense mutation in the *NFKB2* gene presenting in Addisonian crisis associated with intracranial hypertension following ten days of non-specific symptoms of cortisol deficiency [3]. An incidental finding of panhypogammaglobulinaemia led to further immune evaluation which revealed impaired B cell differentiation and function. Our patient had evidence of impaired B cell function and a history of frequent URTIs in early childhood years.

Our experience highlights the importance of identifying the underlying aetiology for a child presenting in an Addisonian crisis. Symptoms may be vague and gradual, and are often overlooked until a stressor precipitates a crisis (Table 2)[6]. Glucocorticoid deficiency, the underlying defect responsible for symptoms, can be due to primary adrenal or central dysfunction. Central dysfunction is usually associated with multiple pituitary hormone deficiency and isolated secondary/tertiary axis defects are extremely rare [7].

**Table 2:** Signs and symptoms of an Addisonian crisis [8]

Symptoms
Severe weakness
Syncope
Nausea and vomiting
Abdominal pain
Back pain
Confusion
**Signs**
Hypotension
Abdominal tenderness / guarding
Fever
Altered GCS, delirium

A short Synacthen test was performed two days following presentation after the patient had received high-dose intravenous hydrocortisone for treatment of the Addisonian crisis. Because of the potential for high-dose glucocorticoid therapy to interfere with the Synacthen test result, the test was repeated one month after diagnosis after stopping glucocorticoid therapy for two weeks. Stress doses of glucocorticoids were to be given if the child developed any illness but were not needed. Surprisingly, the repeat Synacthen test results showed extremely low cortisol levels suggesting progressive atrophy of the adrenal zona fasciculata and a lack of any stimulation by ACTH [9]. This may indicate acute evolution of adrenal atrophy, which is supported by the short timeframe of the patient’s symptoms and normal growth.

The diagnostic work-up for children presenting with pseudotumour cerebri is extensive and includes the differential diagnoses cerebral venous sinus thrombosis, craniosynostosis, otitis media, haematological conditions, hypercoagulable states, medications, hypervitaminosis A, obstructive sleep apnoea and chromosomal abnormalities [10, 11]. An association between pseudotumour cerebri and endocrine disorders is recognised, but primary adrenal insufficiency has rarely been reported in children presenting with raised intracranial pressure [12]. ‘Idiopathic intracranial hypertension’ is rare in prepubertal children, and therefore careful evaluation for an underlying cause and including cortisol in diagnostic investigations are recommended.

The pathophysiology of raised intracranial pressure in corticosteroid deficiency is poorly understood. The enzyme 11ß-hydroxysteroid dehydrogenase type 1, which converts inactive cortisone to cortisol, is expressed in the choroid plexus suggesting a role for cortisol in the regulation of CSF production [13]. Furthermore withdrawal of corticosteroid may reduce the absorption of CSF and increase resistance to CSF flow [14]. The patient’s symptoms recurred when acetazolamide was ceased despite hydrocortisone replacement, indicating longer lasting effects of corticosteroid deficiency on CSF autoregulation, and/or mild persistence of her transverse venous stenosis at the time of weaning.

The patient had evidence of impaired B cell differentiation and function with frequent URTIs in early childhood years. Her clinical phenotype may evolve over time, and patients harbouring mutations in *NFKB2* require longitudinal surveillance not only for infective but also autoimmune complications.

## Conclusion

We report the case of a four-year-old girl with DAVID syndrome presenting in Addisonian crisis associated with pseudotumour cerebri. The case demonstrates the importance of measuring cortisol in children presenting with features of pseudotumour cerebri and that immunoglobulins should be tested in individuals with isolated central hypocortisolaemia. A diagnosis of DAVID syndrome should be considered in all patients presenting with low cortisol and neurological symptoms.

## Data Availability

Data is publically available in ClinVar database, accession number VCV000065385.24.

## Abbreviations

DAVID syndrome: Deficit in anterior pituitary function with variable immune deficiency syndrome
ACTH: Adrenocorticotrophic hormone
CSF: Cerebrospinal fluid
URTIs: Upper respiratory tract infections
CVID: Common variable immunodeficiency

## References

[1] Tovo PA, Lala R, Martino S, Pastorelli G, De Sanctis C (1991). Isolated adrenocorticotropic hormone deficiency associated with common variable immunodeficiency. Eur J Pediatr.

[2] Quentien MH, Delemer B, Papadimitriou DT, Souchon PF, Jaussaud R, Pagnier A (2012). Deficit in Anterior Pituitary Function and Variable Immune Deficiency (DAVID) in Children Presenting with Adrenocorticotropin Deficiency and Severe Infections. J Clin Endocrinol Metab.

[3] Klemann C, Camacho-Ordonez N, Yang L, Eskandarian Z, Rojas-Restrepo JL, Frede N (2019). Clinical and Immunological Phenotype of Patients With Primary Immunodeficiency Due to Damaging Mutations in NFKB2. Front Immunol.

[4] Lal RA, Bachrach LK, Hoffman AR, Inlora J, Rego S, Snyder MP (2017). A Case Report of Hypoglycemia and Hypogammaglobulinemia: DAVID Syndrome in a Patient With a Novel NFKB2 Mutation. J Clin Endocrinol Metab.

[5] Nogueira M, Pinheiro M, Maia R, Silva RS, Costa C, Campos T (2020). Symptomatic hypoglycemia in a child with common variable immunodeficiency: Deficient anterior pituitary with variable immune deficiency (DAVID) syndrome. Clin Pediatr Endocrinol.

[6] Shulman DI, Palmert MR, Kemp SF, for the Lawson Wilkins Drug and Therapeutics Committee (2007). Adrenal Insufficiency: Still a Cause of Morbidity and Death in Childhood. Pediatrics.

[7] Charmandari E, Nicolaides NC, Chrousos GP (2014). Adrenal insufficiency. The Lancet.

[8] Dineen R, Thompson CJ, Sherlock M (2019). Adrenal crisis: prevention and management in adult patients. Ther Adv Endocrinol Metab.

[9] Gonzálbez J, Villabona C, Ramón J, Navarro MA, Giménez O, Ricart W, et al. Establishment of reference values for standard dose short synacthen test (250 μg), low dose short synacthen test (1 μg) and insulin tolerance test for assessment of the hypothalamo-pituitary-adrenal axis in normal subjects. Clin Endocrinol. 2000;53(2):199–204. 10.1046/j.1365-2265.2000.01028.x 10931101

[10] Avery RA, Shah SS, Licht DJ, Seiden JA, Huh JW, Boswinkel J (2010). Reference range for cerebrospinal fluid opening pressure in children. N Engl J Med.

[11] Friedman DI, Liu GT, Digre KB (2013). Revised diagnostic criteria for the pseudotumor cerebri syndrome in adults and children. Neurology.

[12] Shah V, Hoyos-Martinez A, Horne VE (2020). Association of Adrenal Insufficiency With Pediatric Pseudotumor Cerebri Syndrome. JAMA Ophthalmol.

[13] Shenouda S, Al-Farawi K, Dolan J, Flesher SL (2018). Idiopathic intracranial hypertension as a presenting sign of adrenal insufficiency. SAGE Open Med Case Rep.

[14] Johnston I, Gilday DL, Hendrick EB (1975). Experimental effects of steroids and steroid withdrawal on cerebrospinal fluid absorption. J Neurosurg.

